# Talc seromadesis in patients with chronic seroma formation after breast surgery

**DOI:** 10.1186/s40064-015-1648-5

**Published:** 2016-01-04

**Authors:** Coriene J. L. M. Catsman, Martinus A. Beek, Arjen M. Rijken

**Affiliations:** Department of Surgery, Amphia Hospital Breda, Molengracht 21, 4818 CK Breda, The Netherlands

**Keywords:** Talc seromadesis, Breast cancer, Chronic seroma formation

## Abstract

**Background:**

Seroma formation, after breast surgery is a commonly seen complication with an incidence ranging from 10 to 85 %. In general, though seroma usually disappears within several weeks, some patients are troubled with chronic seroma. Seroma predisposes to infection leading to wound related complications and may also delay adjuvant chemoradiation, leading to less effective breast cancer treatment.

**Case description:**

A 52-years-old Dutch woman presenting with a pT3N1M0 infiltrating lobular breast carcinoma received neo-adjuvant chemotherapy, following modified radical mastectomy with sentinel node procedure. She suffered from chronic seroma formation, delaying adjuvant radiation therapy. We successfully performed talc seromadesis to eliminate seroma formation. During six-month follow-up, no seroma was detected.

**Discussion and evaluation:**

Talc seromadesis after breast surgery is a rarely described in literature. In order to develop this technique further research aimed to evaluate its effectiveness on safety and possible later breast reconstruction must be carried out.

**Conclusion:**

Talc seromadesis may be a safe, cost effective and minimally invasive solution to treat chronic seroma after breast surgery.

## Background

Seroma formation after breast surgery is a commonly seen complication with an incidence ranging from of 10 to 85 %. The incidence of seroma formation has been shown to correlate with age, hypertension and breast size, and it may be operator and technique dependent (Kuroi et al. 2005). In general, though seroma usually disappears within several weeks, some patients are troubled with chronic seroma requiring continued seroma aspiration. It is assumed that seroma formation is caused by insufficient lymph drainage. Seroma predisposes to infection leading to wound related complications and may also delay adjuvant chemoradiation, leading to less effective breast cancer treatment (McCaul et al. 2000).

Seroma formation is a commonly seen complication following breast surgery in our surgical center. In this case report we describe a patient with chronic seroma formation after breast surgery who is successfully treated with talc seromadesis.

## Case description

A 52-years-old Dutch woman presenting with a pT3N1M0 infiltrating lobular breast carcinoma received neo-adjuvant chemotherapy, following modified radical mastectomy with sentinel node procedure. She suffered from chronic seroma formation. During initial surgery two drains were placed in both the mammary region and the axillary region. The drain positioned in the mammary region was removed 3 days after surgery because of minor liquid collection. The drain positioned in the axillary region was removed 12 days after surgery because of production of less than 30 mL a day. Seroma was first detected 14 days after surgery, being aspirated at day 24 after surgery. During a period of two and a half months seroma was aspirated nine times. Because of recurrent seroma formation adjuvant radiation therapy was delayed.

In order to continue breast cancer treatment, we tried to find an effective solution to eliminate seroma formation. Currently, there is no evidence based standardized procedure for treating chronic seroma. Several techniques to solve chronic seroma formation such as surgical closure of the dead space between skin and thorax wall (O’Dwyer et al. 1991), talc seromadesis of the seroma cavity (Saeb-Parsy et al. 2006), tetracycline sclerotherapy (Taghizadeh et al. 2008) and wound closure with a quilting technique (Sajid et al. 2011).

In consultation with the patient and our breast surgery team we decided to perform talc seromadesis because this procedure is described as an effective solution to treat seroma formation (Saeb-Parsy et al. 2006).

## Technique description

**Fig. 1:**
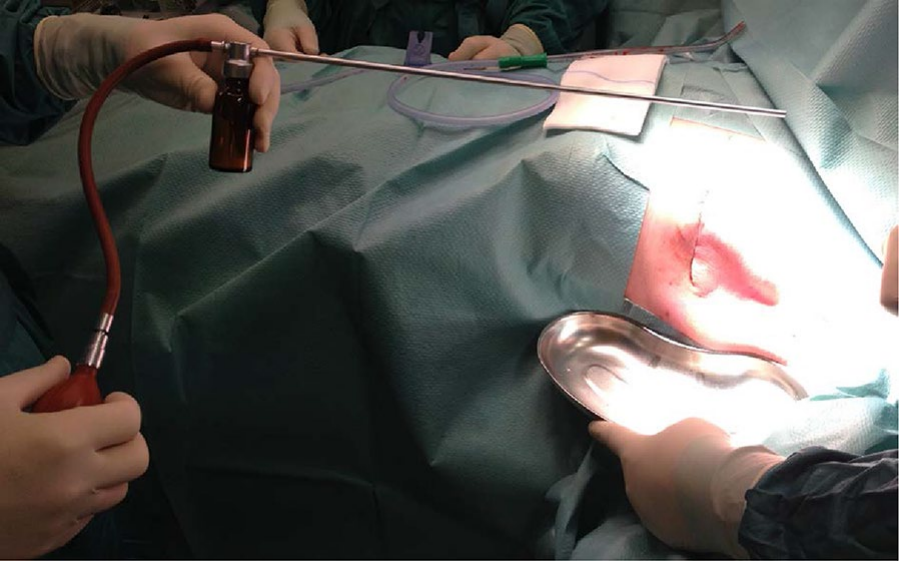
Talc insuflatting device

**Fig. 2:**
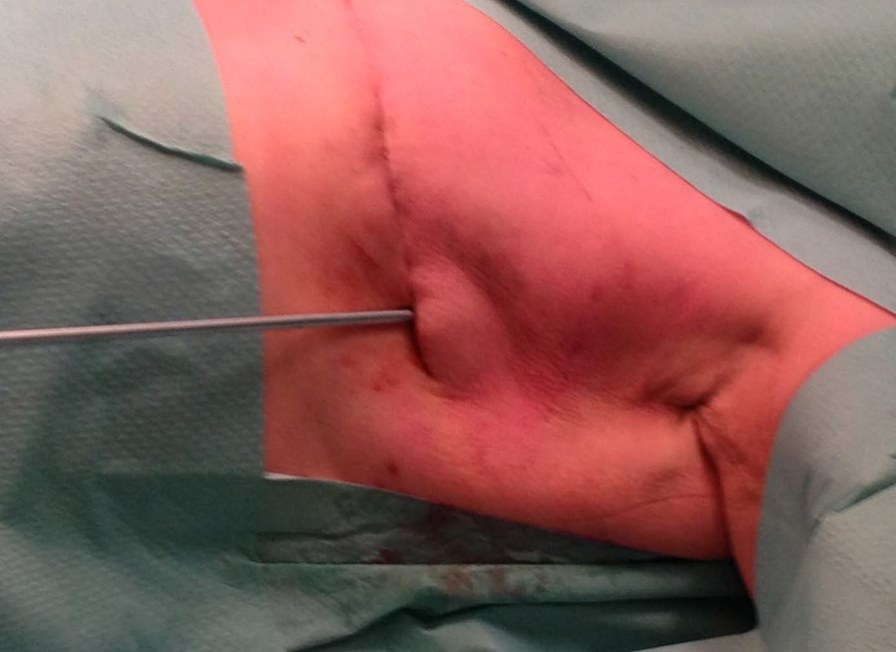
Talc insuflattion into operation wound

Under full anesthesia, a 5 cm long lateral incision was made in the line of the previous operation wound. Sterilized dry talc powder was insufflated into the wound (Figs. 1 and 2). A drain was positioned and a compression bandage was assembled.

## Result

**Fig. 3:**
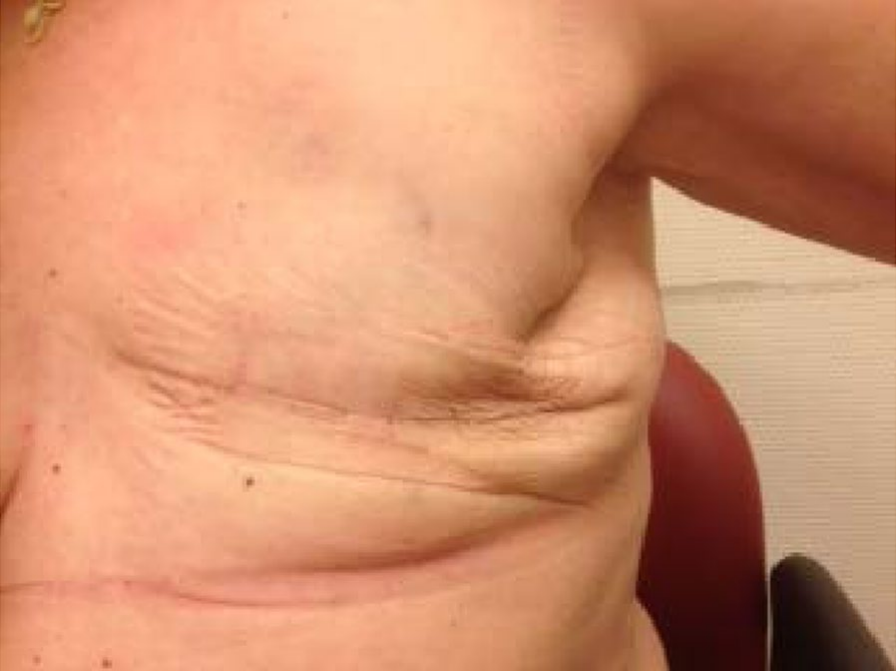
Result one-month after talc seromadesis

The compression bandage and drain were removed 1 week after the procedure and collected a total of 145 mL. Both the procedure and recovery of the patient were without complications. Three weeks after talc seromadesis, the patient was fully recovered (Fig. 3). During 6 month follow-up, no seroma recurred. The patient was able to continue with adjuvant chemoradiation.

## Discussion and evaluation

Talc seromadesis is inspired by pleurodesis and is a widely accepted technique to treat pleural effusion and recurrent pneumothorax. In talc seromadesis, talc is used as a sclerotic agent which induces a fibrotic reaction, causing adhesion between skin and thoracic wall (Saeb-Parsy et al. 2006, Rodriguez-Panadero and Antony 1997).

Talc seromadesis after breast surgery is a poorly described subject in literature. It appears to be a safe, cost-effective and minimal invasive method solution to treat chronic seroma formation after breast surgery. In order to develop this technique further research aimed to evaluate its effectiveness on safety and possible later breast reconstruction must be carried out.

## Conclusion

Talc seromadesis may be a safe, cost effective and minimally invasive solution to treat chronic seroma formation after breast surgery.

## Consent

Written informed consent was obtained from the patient for publication of this case report and any accompanying images. A copy of the written consent is available for review by the Editor of this journal.

## Abbreviation

pTNM: pathology/tumor/nodus/metastasis
